# Knee sleeves improve gait symmetry during fast walking in older adults

**DOI:** 10.3389/fbioe.2024.1394314

**Published:** 2024-07-17

**Authors:** Takuma Inai, Shoma Kudo, Wakako Tsuchida, Masahiro Fujimoto

**Affiliations:** Health and Medical Research Institute, National Institute of Advanced Industrial Science and Technology (AIST), Takamatsu, Kagawa, Japan

**Keywords:** walking, harmonic ratio, acceleration, inertial measurement unit, motion capture

## Abstract

Knee sleeves are commonly used to address knee-related concerns, particularly in older individuals. Although previous studies have demonstrated their efficacy in improving gait and functional outcomes in knees with pathological conditions, the effectiveness of knee sleeves for improving gait characteristics in healthy older adults remains unclear. The harmonic ratio (HR), an index for assessing gait symmetry commonly used to discriminate between individuals with different functional levels, can be used to detect alterations in gait characteristics. This study investigated the effects of knee sleeves on gait symmetry in healthy older adults. Sixteen healthy community-dwelling older adults walked barefoot with and without knee sleeves at normal and fast speeds. Gait symmetry indices (HR and improved HR [iHR]) and spatiotemporal gait parameters were compared under different conditions. A significant interaction between knee condition and walking speed was observed for mean iHR in the anteroposterior direction (*p* = 0.006). A significant simple main effect of knee condition was found during fast walking, with a larger iHR with knee sleeves than without (*p* = 0.002). In the condition without knee sleeves, the iHR was significantly lower during fast walking than during normal walking (*p* = 0.035). Furthermore, a significant main effect of knee condition was observed for the variability of iHR in the anteroposterior direction, with a smaller variability when walking with knee sleeves than when walking without (*p* = 0.006). These results suggest that knee sleeves may enhance gait symmetry along the anteroposterior direction, particularly during fast walking, where symmetry disruption is more likely than walking at a comfortable pace. A significant reduction in gait symmetry variability also suggests a stabilizing effect on gait dynamics. These findings provide the first evidence supporting the efficacy of knee sleeves for improving gait symmetry. The use of knee sleeves could be a valuable option for restoring disrupted gait symmetry during fast walking, with potential implications for reducing the risk of falls.

## 1 Introduction

Knee sleeves are commonly used by individuals experiencing knee-related issues, particularly older individuals, in various contexts, including daily activities and clinical settings. Previous studies have demonstrated the effectiveness of knee sleeves in alleviating knee pain (Bryk et al., 2011; Cudejko et al., 2018; Mohd Sharif et al., 2019; Dzidotor et al., 2023) and influencing gait parameters (Schween et al., 2015; Mohd Sharif et al., 2019). However, most observed enhancements in gait and functional aspects appear to be limited to knees with pathological conditions, such as knee osteoarthritis (Bryk et al., 2011; Collins et al., 2011; Collins et al., 2014; Schween et al., 2015; Mohd Sharif et al., 2019) and anterior cruciate ligament reconstruction (Sole et al., 2022). Consequently, the biomechanical effect of knee sleeves during gait in healthy populations remains poorly understood (Ko et al., 2017). Current research appears to lack sufficient quantitative evidence to substantiate the efficacy of knee sleeves with respect to gait characteristics, particularly in healthy older adults.

The harmonic ratio (HR), a quantitative assessment metric for evaluating the quality of walking, has been widely used to discriminate between populations of different ages and during different functions (Menz et al., 2003a; Lowry et al., 2012; Doi et al., 2013; 2015; Bisi et al., 2014; Brodie et al., 2015; Bisi and Stagni, 2016; Howcroft et al., 2016; Pau et al., 2017; 2020; Iijima et al., 2019; Wada et al., 2019; Leban et al., 2020; Conway et al., 2021; Castiglia et al., 2022; Igarashi et al., 2023). The HR index is calculated based on the harmonic content of the acceleration signals, which are generally acquired at a lower trunk level through spectral analysis. It assesses the symmetry of the body’s acceleration pattern within a gait cycle (a stride) (Pasciuto et al., 2017). Since a stride consists of two steps, the acceleration pattern should inherently repeat in multiples of two within any given stride. Consequently, acceleration patterns in both the anteroposterior (AP) and vertical directions are biphasic for each stride, primarily dominated by the second harmonic (Menz et al., 2003b). On the other hand, mediolateral (ML) acceleration differs from AP or vertical accelerations, as the fundamental ML pattern is limb-dependent (left or right) and thus repeats only once per stride. Therefore, ML accelerations are primarily dominated by odd rather than even harmonics (Menz et al., 2003b). Hence, a perfectly symmetrical gait exclusively results in even harmonics along the AP and vertical directions, whereas only odd harmonics are present in the ML direction (Roche et al., 2013). HR is defined as the ratio between the sum of the amplitudes of these harmonics, which are considered intrinsic to gait, and the extrinsic harmonics that lead to deviations from the ideal gait (Roche et al., 2013; Pasciuto et al., 2017). Using this index, previous studies have reported various factors affecting gait symmetry, such as age (Lowry et al., 2012; Bisi et al., 2014; Bisi and Stagni, 2016; Leban et al., 2020), fall risk (Menz et al., 2003a; Doi et al., 2013; Brodie et al., 2015; Howcroft et al., 2016), cognitive impairment (Pau et al., 2020), white matter lesions (Doi et al., 2015), total hip arthroplasty (Wada et al., 2019), knee pain and disability in knee osteoarthritis (Iijima et al., 2019), multiple sclerosis (Pau et al., 2017), malnutrition (Misu et al., 2017), and the effectiveness of rehabilitation in Parkinson’s disease (Conway et al., 2021; Castiglia et al., 2022) or stroke (Igarashi et al., 2023). These studies have clearly demonstrated that the HR index is a sensitive measure for identifying individuals with functional limitations or pathological conditions, suggesting that gait symmetry is altered in these populations. However, to the best of our knowledge, no study has investigated the effects of knee sleeves on gait symmetry in healthy individuals. Whether the use of knee sleeves alters gait symmetry, even in healthy older adults, remains unclear.

The purpose of this study was to examine the effects of knee sleeves on gait symmetry in healthy older adults, both at a comfortable pace and at a faster pace, to challenge motor and postural control. We hypothesized that the application of knee sleeves would improve gait symmetry, particularly under challenging fast walking conditions.

## 2 Methods

### 2.1 Participants

Sixteen community-dwelling healthy older adults participated (eight males and eight females, age: 71.8 ± 3.4 years, height: 1.59 ± 0.09 m, body mass: 55.5 ± 8.5 kg). Participants were excluded if they required walking aids, had undergone surgery for trauma or orthopedic conditions, had neurological disorders, or were professional athletes. All participants reported no history of neurological pathology, head trauma, cerebrovascular accident, vestibular dysfunction, or visual impairment uncorrectable by lenses. This study adhered to the principles of the Declaration of Helsinki. The study protocol was approved by the Ethics Committee of AIST, Japan (IRB number: hi2023-534). All participants provided written informed consent before participation.

### 2.2 Instrumentation and data collection

**FIGURE 1:**
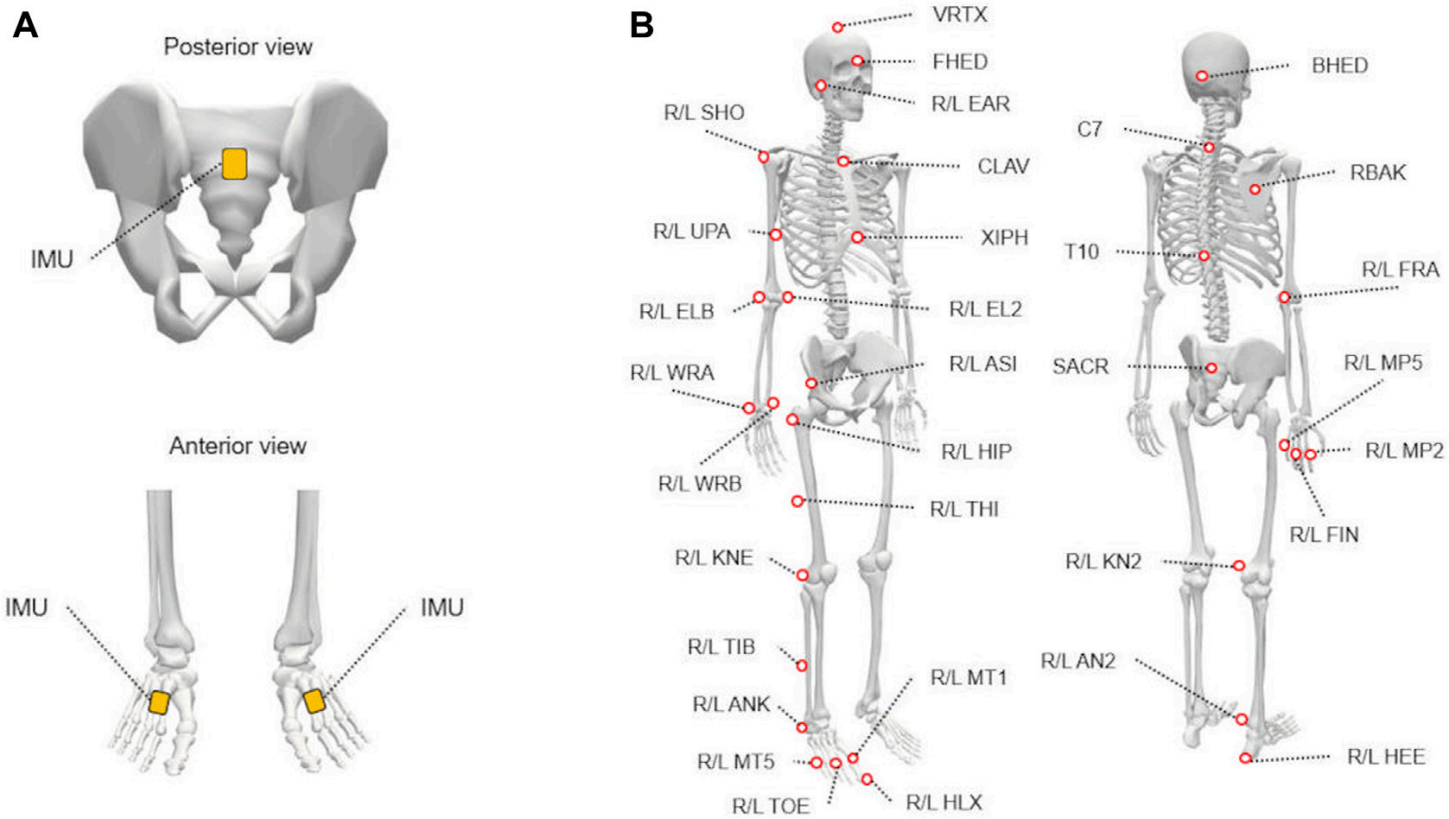
The placements of inertial measurement units (IMUs) and reflective markers. **(A)** Three IMUs were attached to the sacrum and dorsum of the feet. **(B)** Fifty-seven reflective markers were attached to various landmarks on the body (see Supplementary Material S1 for abbreviations). This figure was created using OpenSim (Delp et al., 2007; Seth et al., 2018).

An inertial measurement unit (IMU) (XsensDOT; Movella Inc., Henderson, NV, United States) was placed on the sacrum and dorsum of each foot (Figure 1A). Acceleration data were collected at 120 Hz. The sacral acceleration was used to calculate the index of gait symmetry, whereas the angular velocities of the feet were used to determine the timing of the heel-strike instant.

Fifty-seven reflective markers were placed on each participant’s bony landmarks (Kobayashi et al., 2019) (Figure 1B). Three-dimensional marker trajectories were collected using a 10-camera motion capture system (MAC3D; Motion Analysis Corp., Santa Rosa, CA, United States) at 200 Hz. Trajectories were smoothed using a fourth-order Butterworth filter with an 8-Hz cut-off frequency (Chang et al., 2008; Schmitz et al., 2014). The center of the hip joint was estimated using the method described by Bell et al. (1987), Bell et al. (1990). The centers of the knee and ankle joints were defined as the midpoints of the femoral epicondyles and malleoli, respectively (Inai et al., 2021). Marker trajectories and joint centers were used to calculate the gait parameters.

### 2.3 Protocol

**FIGURE 2:**
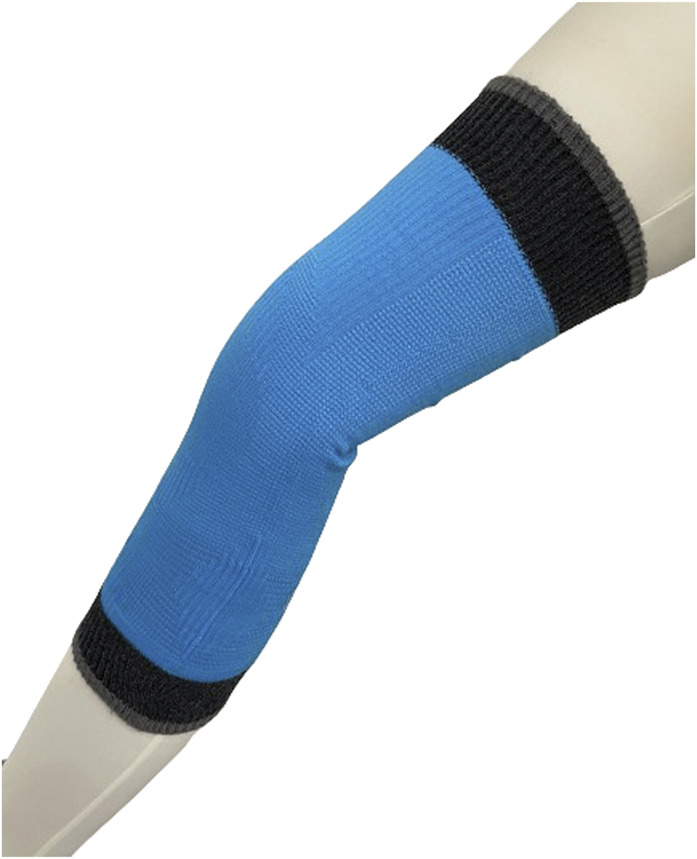
Knee sleeves used in this study. Each participant wore knee sleeves on both knees.

Participants walked barefoot back and forth along a 15-m straight pathway five times at two different walking speeds (Normal [Norm] and Fast), both with and without knee sleeves (KS and CO). They were instructed to walk at their preferred, comfortable speed in the Norm speed condition. For the Fast speed condition, they were asked to walk as fast as possible. They wore knee sleeves (Actcyc Walk [ACT-80], Kagawa Seamless Inc., Kagawa, Japan; Figure 2) on both sides during the KS condition and walked without wearing them during the CO condition. A knee sleeve with a standard wraparound design without a hinge was selected, considering the need for regular use by the participants. Given the potential impact on subsequent comfortable walking speeds and patterns when performing fast walking first (Zhang et al., 2022), the Norm speed condition was followed by the Fast speed condition. The order of the CO and KS conditions was counterbalanced among the participants.

### 2.4 Data analysis

#### 2.4.1 Gait symmetry

The HR (no unit) and improved HR (iHR; unit: %) were calculated using frequency analysis to evaluate the symmetry of the acceleration pattern in the AP, ML, and V directions (Roche et al., 2013; Pasciuto et al., 2017). The sacral IMU accelerations were used for the calculations. Since filtering can potentially cause harmonic distortion (Pasciuto et al., 2017), the raw signals were left unfiltered. Higher values in the HR and iHR indices indicate greater symmetry. The harmonic contents of the acceleration signal, categorized as “in phase” (intrinsic harmonics), were compared to those categorized as “out of phase” (extrinsic harmonics). Since a perfectly symmetrical gait exhibits even harmonics along the AP and V directions, whereas only odd harmonics are present in the ML direction, we considered even harmonics as intrinsic for the AP and V accelerations, and odd harmonics as intrinsic for the ML acceleration (Roche et al., 2013; Pasciuto et al., 2017). The HR was then calculated by dividing the sum of the amplitudes of the intrinsic harmonics by the sum of the amplitudes of the extrinsic harmonics. Specifically, a perfectly symmetrical gait would yield only even harmonics in the AP and V directions and only odd harmonics in the ML direction as “in phase” (intrinsic harmonics) (Roche et al., 2013; Pasciuto et al., 2017). The iHR is an improved index that demonstrates greater robustness and reliability than the traditional HR (Pasciuto et al., 2017). It was proposed to address the following limitations of the HR index: (1) its significant variability in highly symmetrical gait due to the minor contribution of extrinsic harmonics (in the denominator), (2) its unintuitive interpretation, and (3) its lack of mathematical rigor. Derived from the power of the harmonics involved and expressed as a percentage, the iHR allows for a more intuitive understanding and interpretation. The formulas for calculating HR and iHR are as follows (Pasciuto et al., 2017):

$$HR=\frac{\sum_{j=1}^{k}A_{I}^{j}}{\sum_{j=1}^{k}A_{E}^{j}},$$

$$iHR=\frac{\sum_{j=1}^{k}{\left(A_{I}^{j}\right)}^{2}}{\sum_{j=1}^{k}{\left(A_{I}^{j}\right)}^{2}+\sum_{j=1}^{k}{\left(A_{E}^{j}\right)}^{2}}\cdot 100,$$

where k represents the number of intrinsic (or extrinsic) harmonics ( k = 10). Therefore, the total number of considered harmonics is 20. $A_{I}^{j}$ and $A_{E}^{j}$ indicate the amplitudes of the j th intrinsic and extrinsic harmonic, respectively.

This calculation involved 20 harmonics and 20 strides (four strides per straight-path round trip) (Pasciuto et al., 2017). We calculated the mean and standard deviation (SD) of the HR and iHR in the AP, ML, and vertical directions using the 20 strides for each participant. These values were used to represent the mean and within-subject variability. To define a gait cycle (one stride), the heel-strike timing was identified by detecting the zero-crossing point using the foot angular velocity measured by the IMU in the sagittal plane (Jung et al., 2021; Shi et al., 2023). We utilized our original codes written in Scilab 6.1.1 (Dassault Systèmes, Vélizy-Villacoublay, France) for data analysis, including the extraction of harmonics and calculation of the HR and iHR.

#### 2.4.2 Gait parameters

Spatiotemporal gait parameters (walking speed, peak walking velocity and acceleration, step length, stride length, walk ratio, cadence, step time, stride time, stance time, swing time, and minimum toe clearance) and joint angles (peak hip flexion and extension angles, peak knee flexion angle, peak ankle dorsiflexion, and plantarflexion angles, and their range of motion) were calculated based on three-dimensional motion capture data.

Walking speed was calculated using stride time and AP displacement of the whole-body center of mass (COM). Using body parameters, the COM was calculated as the weighted sum of 15 body segments, including the head and neck, trunk, pelvis, upper arms, forearms, hands, thighs, shanks, and feet (Robertson et al., 2008). Walking velocity and acceleration were calculated as the first and second derivatives of AP COM displacement, respectively. Step and stride lengths were calculated as the AP distances between the heel markers from the first to the second heel strike of the contralateral and ipsilateral legs, respectively. Both parameters were normalized to body height. The walk ratio was calculated by dividing the step length by the cadence (Lindemann et al., 2014; Peng et al., 2020). Cadence was calculated using stride time (i.e., 60 × 2/stride time). The stride, stance, and swing times were determined using the heel-strike and toe-off timings. Heel-strike and toe-off timings were detected using midfoot vertical velocity (O’Connor et al., 2007). The hip, knee, and ankle joint angles were computed using the local coordinate systems of the pelvis, thigh, shank, and foot in the ipsilateral leg (Inai et al., 2023). The sagittal plane range of motion for these joints during the gait cycle was also calculated. Minimum toe clearance was defined as the minimum value of the toe marker in the vertical direction during 30%–90% of the swing phase (Yamagata et al., 2019). It was then normalized to body height (Ullauri et al., 2019; Inai et al., 2023).

### 2.5 Statistical analysis

Two-way repeated-measures analysis of variance (ANOVA) with speed (Norm and Fast) and condition (CO and KS) was used to examine the main and interaction effects. When an interaction was confirmed, a paired *t*-test was used to determine simple main effects. The index of effect size (*d* for pairwise comparison; partial eta-squared, $\eta_{p}^{2}$ for ANOVA) was reported as *p*-values. Small, medium, and large effects for Cohen’s *d* were defined as 0.2–0.5, 0.5–0.8, and >0.8, respectively (Cohen, 1992). Small, medium, and large effects for $\eta_{p}^{2}$ were defined as 0.01–0.06, 0.06–0.14, and >0.14, respectively (Cohen, 1992). Statistical analyses were performed using R language 4.3.0 (R Core Team, Vienna, Austria). Statistical significance was set at *p* < 0.05 for all comparisons.

## 3 Results

### 3.1 Gait symmetry

**FIGURE 3:**
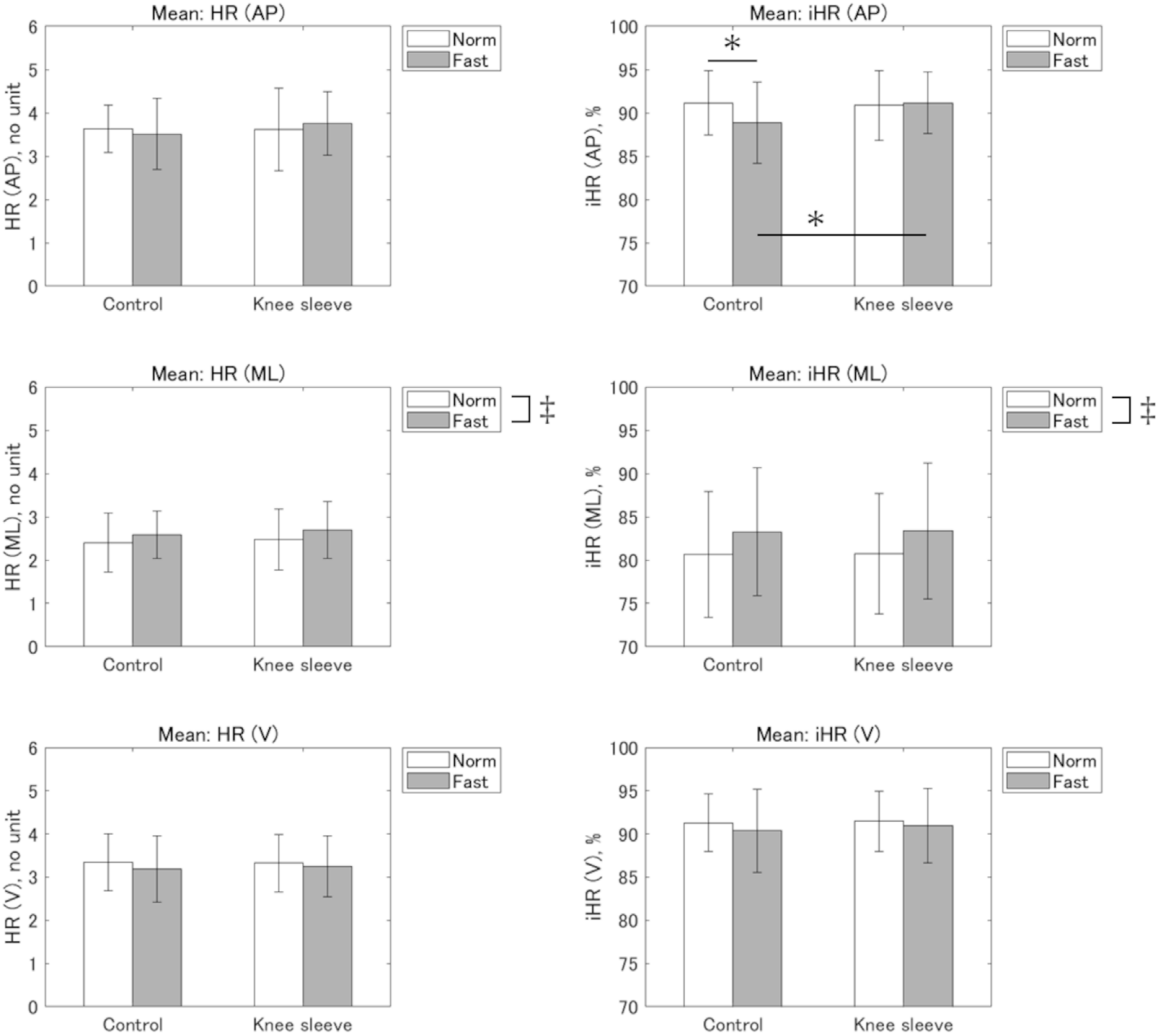
Means of harmonic ratio (HR) and improved HR (iHR). AP: antero-posterior; ML: medio-lateral; V: vertical. * Significant interaction (*p* < 0.05). ‡ Significant main effect of speed (*p* < 0.05).

Figure 3 shows mean HR and iHR. A significant interaction between speed and condition was observed for the mean iHR in the AP direction (*p* = 0.006, $\eta_{p}^{2}$ = 0.40). A simple main effect of condition was found for Fast speed. A pairwise comparison revealed that iHR was significantly larger in the KS condition compared with the CO condition (*p* = 0.002, *d* = 0.53; CO: 88.9% ± 4.8% vs KS: 91.2% ± 3.6%). A simple main effect of speed was also observed under the CO condition. A pairwise comparison revealed that iHR was significantly smaller in the Fast speed condition compared to the Norm speed condition (*p* = 0.035, *d* = 0.52; Norm: 91.2% ± 3.9% vs Fast: 88.9% ± 4.8%). No significant interactions were observed for the other gait symmetry indices.

Significant main effects of speed were observed for mean HR and iHR in the ML direction (*p* = 0.019, $\eta_{p}^{2}$ = 0.32, and *p* = 0.038, $\eta_{p}^{2}$ = 0.26, respectively). The HR and iHR were both significantly larger in the Fast speed condition compared to the Norm speed condition (Norm: 2.4 ± 0.7 vs Fast: 2.6 ± 0.6 and Norm: 80.7% ± 7.2% vs Fast: 83.3% ± 7.8%, respectively).

**FIGURE 4:**
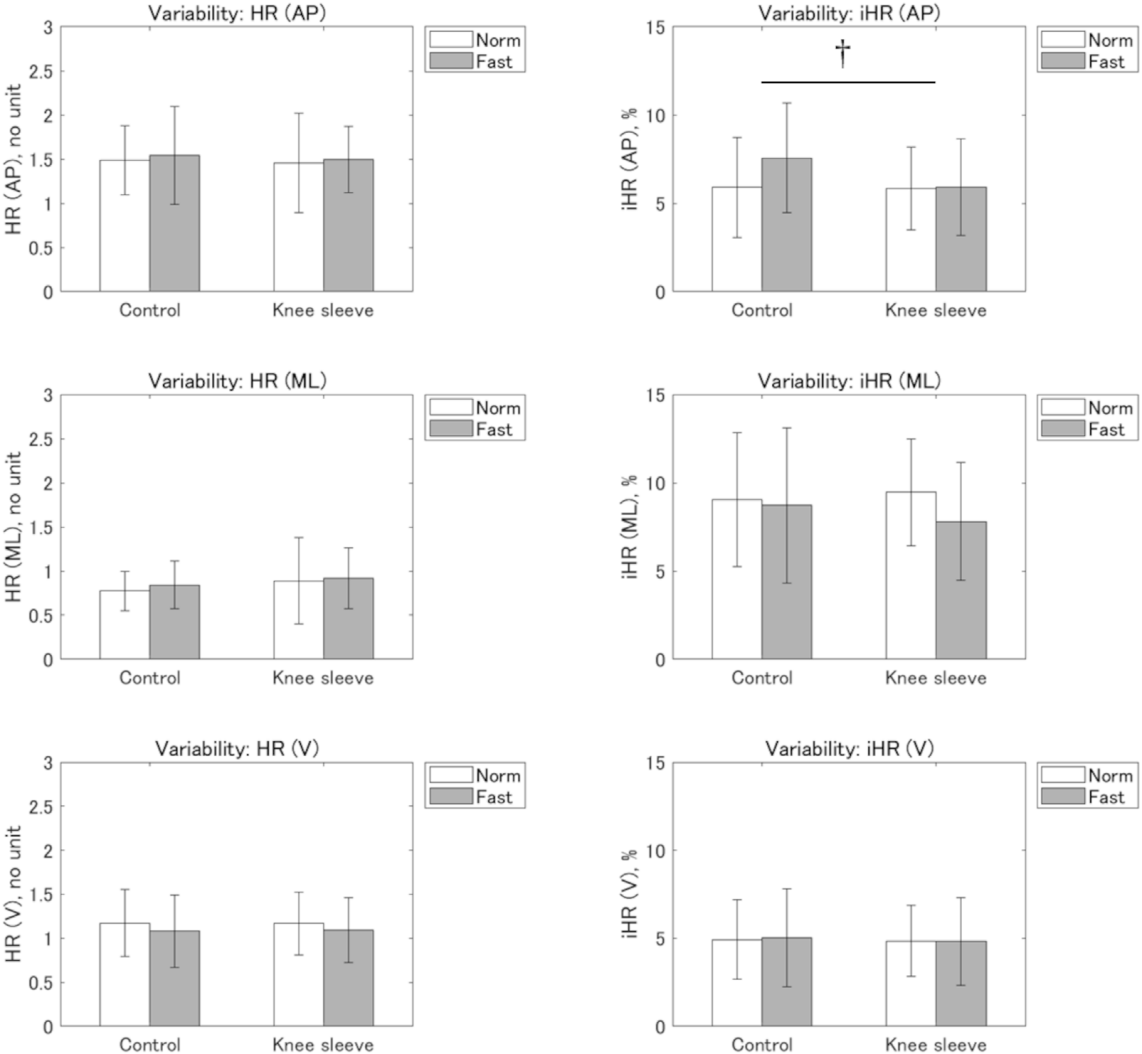
Variabilities in the harmonic ratio (HR) and improved HR (iHR). AP: antero-posterior; ML: medio-lateral; V: vertical. † Significant main effect of condition (*p* < 0.05).

Figure 4 shows the within-subject variability of the HR and iHR. No significant interactions were observed for any of the gait symmetry indices. A significant main effect of condition was observed for the variability of iHR in the AP direction (*p* = 0.006, $\eta_{p}^{2}$ = 0.41). The variability was smaller in the KS condition compared with the CO condition (CO: 6.7% ± 3.1% vs KS: 5.9% ± 2.6%).

### 3.2 Gait parameters

Table 1 shows the mean gait parameters of the participants (see Supplementary Material S2 for details). No significant interactions were observed for any parameters. Significant main effects of the condition were observed for the mean swing time, peak hip flexion angle, range of motion of the hip and knee joints, and minimum toe clearance (*p* ≤ 0.024). Significant main effects of speed were observed for the mean walking speed, peak walking speed and acceleration, step and stride lengths, cadence, step and stride times, stance time, swing time, percentages of stance phase and swing phase, and peak hip flexion and ankle plantar flexion angles (*p* ≤ 0.020).

**TABLE 1:** Gait parameters.

Variable	Control		Knee sleeve		Factor					
	Norm	Fast	Norm	Fast	Condition		Speed		Interaction	
	Mean (SD)	Mean (SD)	Mean (SD)	Mean (SD)	*p*-value	Partial η^2^	*p*-value	Partial η^2^	*p*-value	Partial η^2^
Walking speed, m/s	1.32 (0.17)	1.69 (0.21)	1.32 (0.16)	1.71 (0.24)	0.530	0.03	**0.000** ^c^	0.83	0.639	0.02
Peak walking speed, m/s	1.45 (0.17)	1.83 (0.22)	1.45 (0.17)	1.84 (0.25)	0.899	0.00	**0.000** ^c^	0.84	0.654	0.01
Peak walking acceleration, m/s^2^	1.72 (0.40)	2.20 (0.53)	1.67 (0.42)	2.29 (1.05)	0.892	0.00	**0.000** ^c^	0.64	0.575	0.02
Step length, m/HT	0.40 (0.04)	0.44 (0.05)	0.40 (0.04)	0.44 (0.05)	0.419	0.04	**0.000** ^c^	0.63	0.701	0.01
Stride length, m/HT	0.80 (0.08)	0.89 (0.10)	0.80 (0.08)	0.88 (0.10)	0.434	0.04	**0.000** ^c^	0.62	0.651	0.01
Walk ratio, cm/(steps/min)	0.52 (0.06)	0.49 (0.10)	0.51 (0.06)	0.49 (0.10)	0.102	0.17	0.200	0.11	0.639	0.02
Cadence, steps/min	123.7 (11.2)	145.3 (18.6)	124.6 (10.2)	146.7 (21.1)	0.143	0.14	**0.000** ^c^	0.64	0.811	0.00
Step time, s	0.489 (0.046)	0.420 (0.054)	0.484 (0.043)	0.417 (0.058)	0.113	0.16	**0.000** ^d^	0.73	0.846	0.00
Stride time, s	0.978 (0.092)	0.839 (0.107)	0.970 (0.086)	0.834 (0.116)	0.143	0.14	**0.000** ^d^	0.73	0.826	0.00
Stance time, s	0.572 (0.067)	0.470 (0.079)	0.568 (0.065)	0.469 (0.087)	0.542	0.03	**0.000** ^d^	0.73	0.802	0.00
Swing time, s	0.406 (0.026)	0.369 (0.030)	0.401 (0.023)	0.365 (0.031)	**0.002** ^b^	0.47	**0.000** ^d^	0.71	0.880	0.00
Percentage of stance phase, %	58.3 (1.4)	55.8 (2.6)	58.5 (1.5)	55.9 (3.2)	0.187	0.11	**0.000** ^d^	0.57	0.954	0.00
Percentage of swing phase, %	41.7 (1.4)	44.2 (2.6)	41.5 (1.5)	44.1 (3.2)	0.187	0.11	**0.000** ^c^	0.57	0.954	0.00
Peak hip flexion angle, deg^e^	40.9 (6.7)	43.8 (6.9)	40.3 (6.3)	43.3 (6.6)	**0.024** ^b^	0.30	**0.000** ^c^	0.65	0.883	0.00
Peak hip extension angle, deg^e^	−6.2 (6.1)	−6.4 (7.6)	−5.7 (6.2)	−5.8 (8.0)	0.071	0.20	0.897	0.00	0.843	0.00
Peak knee flexion angle, deg	64.9 (2.9)	65.8 (3.5)	64.4 (3.0)	65.1 (3.7)	0.102	0.17	0.050	0.23	0.607	0.02
Peak ankle dorsiflexion angle, deg^f^	14.1 (2.3)	14.0 (2.8)	13.7 (2.5)	13.7 (3.1)	0.121	0.15	0.943	0.00	0.949	0.00
Peak ankle plantar flexion angle, deg^f^	−16.6 (5.7)	−18.5 (6.0)	−17.4 (6.5)	−18.9 (6.5)	0.165	0.12	**0.020** ^d^	0.31	0.566	0.02
Range of motion of hip joint, deg	47.2 (5.9)	50.5 (7.7)	46.2 (5.8)	49.3 (8.2)	**0.004** ^b^	0.44	0.052	0.23	0.803	0.00
Range of motion of knee joint, deg	62.3 (4.4)	62.2 (5.5)	61.4 (4.4)	61.6 (5.2)	**0.001** ^b^	0.55	0.946	0.00	0.559	0.02
Range of motion of ankle joint, deg	30.7 (5.3)	32.5 (5.3)	31.1 (6.3)	32.6 (5.4)	0.492	0.03	0.078	0.19	0.592	0.02
Minimum toe clearance, m/HT	0.0344 (0.0032)	0.0340 (0.0027)	0.0337 (0.0033)	0.0334 (0.0025)	**0.006** ^a^	0.40	0.551	0.02	0.686	0.01

Note: Bold indicates *p* < 0.05. HT, height.

a Control < Knee sleeve.

b Control > Knee sleeve.

c Norm < Fast.

d Norm > Fast.

e Positive numbers indicate hip flexion and negative numbersindicate hip extension.

f Positive numbers indicate ankle dorsiflexion and negative numbers indicate ankle plantar flexion.

Table 2 shows the variability in the gait parameters (see Supplementary Material S3 for details). A significant interaction between the condition and variability of the range of motion of the ankle joint was observed (*p* = 0.046, $\eta_{p}^{2}$ = 0.24). However, no simple main effect was observed in the post-analysis. No significant interactions were observed for any other gait parameters. Significant main effects of the condition were observed for variabilities in peak hip flexion and peak ankle plantar flexion angles (*p* ≤ 0.032). Significant effects of speed were observed for variabilities in walking speed, peak walking speed and acceleration, stride length, cadence, percentages of stance and swing phases, peak hip flexion angle, and range of motion of the knee joint (*p* ≤ 0.044).

**TABLE 2:** Variabilities in gait parameters.

Variable	Control		Knee sleeve		Factor					
	Norm	Fast	Norm	Fast	Condition		Speed		Interaction	
	Mean (SD)	Mean (SD)	Mean (SD)	Mean (SD)	*p*-value	Partial η^2^	*p*-value	Partial η^2^	*p*-value	Partial η^2^
Walking speed, m/s	0.03 (0.01)	0.05 (0.01)	0.03 (0.02)	0.05 (0.02)	0.802	0.00	**0.001** ^c^	0.56	0.736	0.01
Peak walking speed, m/s	0.03 (0.01)	0.05 (0.01)	0.04 (0.02)	0.05 (0.02)	0.713	0.01	**0.003** ^c^	0.45	0.736	0.01
Peak walking acceleration, m/s^2^	0.17 (0.10)	0.35 (0.19)	0.17 (0.04)	0.47 (0.53)	0.384	0.05	**0.008** ^c^	0.39	0.343	0.06
Step length, m/HT	0.011 (0.004)	0.014 (0.005)	0.011 (0.006)	0.014 (0.004)	0.930	0.00	0.050	0.23	0.950	0.00
Stride length, m/HT	0.015 (0.005)	0.019 (0.005)	0.017 (0.010)	0.020 (0.006)	0.401	0.05	**0.028** ^c^	0.28	0.633	0.02
Walk ratio, cm/(steps/min)	0.02 (0.01)	0.02 (0.01)	0.02 (0.01)	0.02 (0.01)	0.702	0.01	0.541	0.03	0.556	0.02
Cadence, steps/min	1.8 (0.6)	2.7 (1.0)	1.9 (0.8)	2.6 (1.1)	0.991	0.00	**0.012** ^c^	0.35	0.488	0.03
Step time, s	0.011 (0.003)	0.011 (0.003)	0.011 (0.004)	0.010 (0.003)	0.731	0.01	0.631	0.02	0.983	0.00
Stride time, s	0.014 (0.005)	0.016 (0.005)	0.015 (0.007)	0.015 (0.007)	0.892	0.00	0.751	0.01	0.466	0.04
Stance time, s	0.012 (0.003)	0.013 (0.004)	0.012 (0.006)	0.012 (0.005)	0.948	0.00	0.702	0.01	0.367	0.05
Swing time, s	0.008 (0.003)	0.007 (0.002)	0.007 (0.002)	0.008 (0.002)	0.922	0.00	0.956	0.00	0.050	0.23
Percentage of stance phase, %	0.69 (0.25)	0.79 (0.18)	0.63 (0.20)	0.81 (0.23)	0.551	0.02	**0.029** ^c^	0.28	0.266	0.08
Percentage of swing phase, %	0.69 (0.25)	0.79 (0.18)	0.63 (0.20)	0.81 (0.23)	0.551	0.02	**0.029** ^c^	0.28	0.266	0.08
Peak hip flexion angle, deg	1.3 (0.5)	1.6 (0.4)	1.2 (0.4)	1.3 (0.4)	**0.003** ^b^	0.45	**0.013** ^c^	0.35	0.097	0.17
Peak hip extension angle, deg	1.4 (0.8)	1.4 (0.7)	1.5 (1.0)	1.5 (0.8)	0.337	0.06	0.888	0.00	0.323	0.07
Peak knee flexion angle, deg	1.7 (0.6)	2.2 (1.2)	1.7 (0.8)	1.8 (0.8)	0.305	0.07	0.082	0.19	0.092	0.18
Peak ankle dorsiflexion angle, deg	2.0 (1.0)	1.8 (1.0)	1.6 (0.7)	1.7 (0.9)	0.071	0.20	0.959	0.00	0.320	0.07
Peak ankle plantar flexion angle, deg	3.0 (2.2)	2.8 (1.1)	2.8 (2.0)	2.4 (1.2)	**0.032** ^b^	0.27	0.309	0.07	0.391	0.05
Range of motion of hip joint, deg	1.6 (0.8)	1.8 (0.7)	1.6 (1.0)	1.6 (0.7)	0.552	0.02	0.643	0.01	0.219	0.10
Range of motion of knee joint, deg	2.1 (0.6)	2.6 (1.3)	2.0 (0.5)	2.3 (0.9)	0.194	0.11	**0.044** ^c^	0.24	0.506	0.03
Range of motion of ankle joint, deg	2.9 (2.0)	3.2 (1.5)	3.0 (2.1)	3.0 (1.6)	0.464	0.04	0.474	0.03	**0.046**	0.24
Minimum toe clearance, m/HT	0.0030 (0.0012)	0.0031 (0.0010)	0.0029 (0.0012)	0.0030 (0.0011)	0.426	0.04	0.446	0.04	0.685	0.01

**Note**: Bold indicates p < 0.05. HT, height.

a Control < Knee sleeve.

b Control > Knee sleeve.

c Norm < Fast.

d Norm > Fast.

## 4 Discussion

This study examined the effects of knee sleeves on gait symmetry. Gait symmetry indices (HR and iHR) and spatiotemporal gait parameters were compared during walking with and without knee sleeves (KS and CO, respectively) under normal and fast walking speed conditions. A significant interaction between knee condition and walking speed was observed for mean iHR in the AP direction. A significant simple main effect of knee condition was found during fast walking, revealing a significantly larger iHR with knee sleeves than without knee sleeves. In the condition without knee sleeves, iHR was significantly lower during fast walking than during normal walking. Furthermore, a significant main effect of knee condition was observed for the variability in iHR in the AP direction, indicating a significantly smaller variability when walking with knee sleeves than without. These results suggest that knee sleeves may enhance gait symmetry, particularly in the AP direction during faster-paced walking, where symmetry might be disrupted compared to walking at a comfortable pace.

Without the use of knee sleeves, the iHR in the AP direction significantly decreased during fast walking compared with normal walking, indicating a reduction in gait symmetry. This finding aligns with previous studies that demonstrated a lower HR along the AP axis at increased walking speeds (Menz et al., 2003b; Latt et al., 2008). This reduction in symmetry index may be associated with an elevated risk of falls during fast walking in older adults. Previous studies have reported an increased likelihood of falls when walking at a faster speed (≥1.3 m/s) (Quach et al., 2011). Similarly, it has been reported that the HR in the AP direction is the highest at the preferred speed (approximately 1.2 m/s) and decreases as the speed increases (Latt et al., 2008). Furthermore, a recognized association exists between gait symmetry and fall risk, with a low HR along the AP axis indicating a higher risk of falls (Menz et al., 2003a; Doi et al., 2015). Walking at a pace faster than one’s comfortable speed appears to challenge the postural control system, potentially disrupting gait symmetry in the AP direction and subsequently increasing the risk of falls.

Based on our results, knee sleeves may be a viable approach to enhance gait symmetry during fast walking. The application of knee sleeves resulted in a significant increase in the iHR in the AP direction during fast walking, reaching a level comparable to that during walking at normal speed. Additionally, a significant decrease in variability in the AP direction was observed with the knee sleeves. These findings suggest that the gait symmetry is enhanced and stabilized when walking faster with knee sleeves. Compression applied to the thigh and shank by the knee sleeves may have contributed to these improvements. Although the underlying mechanisms have not been fully elucidated, previous studies have reported an enhanced joint position sense using knee sleeves (Birmingham et al., 1998; Herrington et al., 2005; Mohd Sharif et al., 2017; Dzidotor et al., 2023), elastic bandages (Perlau et al., 1995), and knee braces (McNair et al., 1996). The heightened sensory input from tactile stimulation as a result of tightness may be a key factor in producing these effects (Mohd Sharif et al., 2017; Dzidotor et al., 2023). Therefore, knee sleeves may induce proprioceptive changes, subsequently modulating gait dynamics and improving the disrupted gait symmetry during fast walking.

The constraint imposed by the knee sleeves may also have contributed to stabilizing the gait dynamics. Walking with knee sleeves resulted in a significant reduction in the range of motion of the knee and hip joints, independent of walking speed. The variability in both peak hip flexion and peak ankle plantar flexion angles also decreased with the knee sleeves. These alterations can be attributed to the compressive effect imposed on the knees by the sleeves, which restricts knee joint movement and influences other joint motions. This restriction seems to have reduced the range and variability of joint motion, which in turn resulted in the improvement of disrupted symmetry. Taken together, knee sleeves may offer a valuable approach for enhancing gait symmetry, potentially reducing the risk of falls during fast walking, possibly owing to their proprioceptive and/or constraining effects on the knee joint.

This study had some limitations. First, the participants were exclusively healthy older adults. Gait kinematics in older adults differ from those in other groups, such as healthy young adults (Chehab et al., 2017; Schloemer et al., 2017) and older adults who have experienced falls (Kobayashi et al., 2014; Inai et al., 2023). As the effect of knee sleeves on gait symmetry in other populations remains unknown, further research is required to investigate their impact across diverse demographics. Second, only one type of knee sleeve was used. It is crucial to recognize that different designs, materials, and tightness characteristics may influence gait symmetry and parameters. Therefore, future studies should explore whether various types of knee sleeves have different effects on these variables. Finally, HR and iHR are global measures and, therefore, cannot explain in which phase of the gait cycle the deviations from symmetry occur or why they happen. Further studies investigating time-series three-dimensional kinematics and kinetics are warranted to elucidate the underlying mechanisms affecting gait symmetry.

In conclusion, this study examined gait symmetry indices and spatiotemporal gait parameters during walking with and without knee sleeves at different walking speeds. Our results indicate that the use of knee sleeves enhances gait symmetry along the AP direction, particularly during fast walking, where symmetry disruption is more likely than when walking at a comfortable pace. Additionally, knee sleeves were found to reduce the variability in gait symmetry across different walking speeds, suggesting a stabilizing effect on gait dynamics. To the best of our knowledge, this is the first study to provide evidence supporting the efficacy of knee sleeves in improving gait symmetry. The use of knee sleeves could be a valuable option for restoring disrupted gait symmetry during fast walking, with potential implications for reducing the risk of falls.

## Data Availability

The datasets presented in this article are not readily available because our IRB approval does not include data sharing. Requests to access the datasets should be directed to TI, takuma.inai@aist.go.jp.
