# Supplementary material for: Knee sleeves improve gait symmetry during fast walking in older adults
Source: Front Bioeng Biotechnol. 2024 Jul 17;12:1394314. doi: 10.3389/fbioe.2024.1394314 (PMC11288883; doi:10.3389/fbioe.2024.1394314)
Supplement: Supplementary file 1 [file DataSheet2.PDF]

Supplementary Table 2. Gait parameters.

Variable	Control, a		Knee sleeve, a		Norm, a		Fast, a	
	Mean	(SD)	Mean	(SD)	Mean	(SD)	Mean	(SD)
Walking speed, m/s	1.50	(0.27)	1.51	(0.28)	1.32	(0.16)	1.70	(0.22)
Peak walking speed, m/s	1.64	(0.27)	1.65	(0.29)	1.45	(0.17)	1.84	(0.23)
Peak walking acceleration, m/s^2	1.96	(0.52)	1.98	(0.85)	1.69	(0.40)	2.25	(0.82)
Step length, m/HT	0.42	(0.05)	0.42	(0.05)	0.40	(0.04)	0.44	(0.05)
Stride length, m/HT	0.85	(0.10)	0.84	(0.10)	0.80	(0.08)	0.88	(0.10)
Walk ratio, cm/(steps/min)	0.51	(0.08)	0.50	(0.08)	0.52	(0.06)	0.49	(0.10)
Cadence, steps/min	134.5	(18.7)	135.6	(19.8)	124.2	(10.5)	146.0	(19.6)
Step time, s	0.454	(0.061)	0.451	(0.061)	0.487	(0.044)	0.418	(0.055)
Stride time, s	0.908	(0.121)	0.902	(0.122)	0.974	(0.088)	0.836	(0.110)
Stance time, s	0.521	(0.089)	0.519	(0.091)	0.570	(0.065)	0.470	(0.082)
Swing time, s	0.388	(0.034)	0.383	(0.033)	0.404	(0.024)	0.367	(0.030)
Percentage of stance phase, %	57.1	(2.5)	57.2	(2.8)	58.4	(1.4)	55.8	(2.9)
Percentage of swing phase, %	42.9	(2.5)	42.8	(2.8)	41.6	(1.4)	44.2	(2.9)
Peak hip flexion angle, deg, b	42.4	(6.9)	41.8	(6.5)	40.6	(6.4)	43.6	(6.7)
Peak hip extension angle, deg, b	-6.3	(6.8)	-5.8	(7.1)	-5.9	(6.0)	-6.1	(7.7)
Peak knee flexion angle, deg	65.4	(3.2)	64.8	(3.3)	64.7	(2.9)	65.5	(3.6)
Peak ankle dorsiflexion angle, deg, c	14.0	(2.5)	13.7	(2.8)	13.9	(2.4)	13.8	(2.9)
Peak ankle plantar flexion angle, deg, c	-17.6	(5.8)	-18.2	(6.4)	-17.0	(6.0)	-18.7	(6.1)
Range of motion of hip joint, deg	48.8	(6.9)	47.7	(7.2)	46.7	(5.8)	49.9	(7.8)
Range of motion of knee joint, deg	62.3	(4.9)	61.5	(4.7)	61.9	(4.3)	61.9	(5.3)
Range of motion of ankle joint, deg	31.6	(5.3)	31.9	(5.8)	30.9	(5.7)	32.5	(5.3)
Minimum toe clearance, m/HT	0.0342	(0.0029)	0.0335	(0.0029)	0.0340	(0.0032)	0.0337	(0.0025)

Note: HT: height. a: This includes both levels of the other factor (e.g., in the case of 'Control', both speeds are included). b: Positive numbers indicate hip flexion and negative numbers indicate hip extension. c: Positive numbers indicate ankle dorsiflexion and negative numbers indicate ankle plantar flexion.
