# Supplementary material for: Knee sleeves improve gait symmetry during fast walking in older adults
Source: Front Bioeng Biotechnol. 2024 Jul 17;12:1394314. doi: 10.3389/fbioe.2024.1394314 (PMC11288883; doi:10.3389/fbioe.2024.1394314)
Supplement: Supplementary file 2 [file DataSheet3.PDF]

Supplementary Table 3. Variabilities in gait parameters.

Variable	Control, a		Knee sleeve, a		Norm, a		Fast, a	
	Mean	(SD)	Mean	(SD)	Mean	(SD)	Mean	(SD)
Walking speed, m/s	0.04	(0.01)	0.04	(0.02)	0.03	(0.01)	0.05	(0.02)
Peak walking speed, m/s	0.04	(0.01)	0.04	(0.02)	0.03	(0.01)	0.05	(0.02)
Peak walking acceleration, m/s^2	0.26	(0.17)	0.32	(0.40)	0.17	(0.08)	0.41	(0.40)
Step length, m/HT	0.012	(0.004)	0.013	(0.005)	0.011	(0.005)	0.014	(0.005)
Stride length, m/HT	0.017	(0.006)	0.018	(0.009)	0.016	(0.008)	0.020	(0.006)
Walk ratio, cm/(steps/min)	0.02	(0.01)	0.02	(0.01)	0.02	(0.01)	0.02	(0.01)
Cadence, steps/min	2.3	(0.9)	2.3	(1.0)	1.9	(0.7)	2.7	(1.1)
Step time, s	0.011	(0.003)	0.011	(0.003)	0.011	(0.003)	0.010	(0.003)
Stride time, s	0.015	(0.005)	0.015	(0.007)	0.015	(0.006)	0.015	(0.006)
Stance time, s	0.012	(0.004)	0.012	(0.005)	0.012	(0.005)	0.013	(0.004)
Swing time, s	0.008	(0.003)	0.008	(0.002)	0.008	(0.003)	0.008	(0.002)
Percentage of stance phase, %	0.74	(0.22)	0.72	(0.23)	0.66	(0.23)	0.80	(0.21)
Percentage of swing phase, %	0.74	(0.22)	0.72	(0.23)	0.66	(0.23)	0.80	(0.21)
Peak hip flexion angle, deg	1.5	(0.5)	1.3	(0.4)	1.3	(0.4)	1.5	(0.4)
Peak hip extension angle, deg	1.4	(0.7)	1.5	(0.9)	1.5	(0.9)	1.4	(0.7)
Peak knee flexion angle, deg	2.0	(1.0)	1.8	(0.8)	1.7	(0.7)	2.0	(1.1)
Peak ankle dorsiflexion angle, deg	1.9	(1.0)	1.7	(0.8)	1.8	(0.9)	1.8	(0.9)
Peak ankle plantar flexion angle, deg	2.9	(1.7)	2.6	(1.6)	2.9	(2.0)	2.6	(1.1)
Range of motion of hip joint, deg	1.7	(0.7)	1.6	(0.9)	1.6	(0.9)	1.7	(0.7)
Range of motion of knee joint, deg	2.4	(1.0)	2.1	(0.8)	2.0	(0.6)	2.5	(1.1)
Range of motion of ankle joint, deg	3.1	(1.7)	3.0	(1.8)	2.9	(2.0)	3.1	(1.5)
Minimum toe clearance, m/HT	0.0031	(0.0011)	0.0030	(0.0012)	0.0029	(0.0012)	0.0031	(0.0011)

Note: HT: height. a: This includes both levels of the other factor (e.g., in the case of 'Control', both speeds are included).
