# Supplementary material for: Knee sleeves improve gait symmetry during fast walking in older adults
Source: Front Bioeng Biotechnol. 2024 Jul 17;12:1394314. doi: 10.3389/fbioe.2024.1394314 (PMC11288883; doi:10.3389/fbioe.2024.1394314)
Supplement: Supplementary file 3 [file DataSheet1.PDF]

Supplementary Table 1. Explanation of marker abbreviations

Marker name	Placement
FHED	Front of head (between the eyebrows)
VRTX	Top of head
BHED	Back of head
R/L EAR	Tragus
C7	Seventh cervical spinous process
CLAV	Top of sternum
XIPH	Lower end of sternum
T10	Tenth thoracic spinous process
RBAK	Right scapula (dummy marker)
R/L ASI	Anterior superior iliac spine
SACR	Sacrum
R/L SHO	Acromion
R/L UPA	Biceps muscle belly
R/L ELB	Lateral epicondyle of humerus
R/L EL2	Medial epicondyle of humerus
R/L FRA	Olecranon
R/L WRA	Radial styloid process
R/L WRB	Ulnar styloid process
R/L FIN	Midpoint between 3rd and 4th metacarpal heads
R/L MP2	Second metacarpal head
R/L MP5	Fifth metacarpal head
R/L HIP	Greater trochanter
R/L THI	Thigh (lateral side)
R/L KNE	Lateral epicondyle of femur
R/L KN2	Medial epicondyle of femur
R/L TIB	Shank (lateral side)
R/L ANK	Lateral malleolus
R/L AN2	Medial malleolus
R/L TOE	Midpoint between 2nd and 3rd metatarsal heads
R/L HEL	Heel
R/L MT1	First metatarsal head
R/L MT5	Fifth metatarsal head
R/L HLX	First toe
